# Siberian larch (*Larix sibirica* Ledeb.) chloroplast genome and development of polymorphic chloroplast markers

**DOI:** 10.1186/s12859-018-2571-x

**Published:** 2019-02-05

**Authors:** Eugeniya I. Bondar, Yuliya A. Putintseva, Nataliya V. Oreshkova, Konstantin V. Krutovsky

**Affiliations:** 1 Laboratory of Forest Genomics, Genome Research and Education Center, Siberian Federal University, 660036 Krasnoyarsk, Russian Federation; 2 Laboratory of Forest Genetics and Selection, V.N. Sukachev Institute of Forest, Siberian Branch of Russian Academy of Sciences, 660036 Krasnoyarsk, Russian Federation; 3 Department of Forest Genetics and Forest Tree Breeding, Georg-August University of Göttingen, Büsgenweg 2, D-37077 Göttingen, Germany; 4 Laboratory of Population Genetics, Vavilov Institute of General Genetics, Russian Academy of Sciences, 119333 Moscow, Russia; 5 Department of Ecosystem Science and Management, Texas A&M University, College Station, TX 77843-2138 USA

**Keywords:** Chloroplast genome, *Larix sibirica*, Sequencing, Siberian larch, SNPs, SSRs

## Abstract

**Background:**

The main objectives of this study were sequencing, assembling, and annotation of chloroplast genome of one of the main Siberian boreal forest tree conifer species Siberian larch (*Larix sibirica* Ledeb.) and detection of polymorphic genetic markers – microsatellite loci or simple sequence repeats (SSRs) and single nucleotide polymorphisms (SNPs).

**Results:**

We used the data of the whole genome sequencing of three Siberian larch trees from different regions - the Urals, Krasnoyarsk, and Khakassia, respectively. Sequence reads were obtained using the Illumina HiSeq2000 in the Laboratory of Forest Genomics at the Genome Research and Education Center of the Siberian Federal University. The assembling was done using the Bowtie2 mapping program and the SPAdes genomic assembler. The genome annotation was performed using the RAST service. We used the GMATo program for the SSRs search, and the Bowtie2 and UGENE programs for the SNPs detection. Length of the assembled chloroplast genome was 122,561 bp, which is similar to 122,474 bp in the closely related European larch (*Larix decidua* Mill.). As a result of annotation and comparison of the data with the existing data available only for three larch species - *L. decidua, L. potaninii var. chinensis* (complete genome 122,492 bp), and *L. occidentalis* (partial genome of 119,680 bp), we identified 110 genes, 34 of which represented tRNA, 4 rRNA, and 72 protein-coding genes. In total, 13 SNPs were detected; two of them were in the *tRNA-Arg* and *Cell division protein FtsH* genes, respectively. In addition, 23 SSR loci were identified.

**Conclusions:**

The complete chloroplast genome sequence was obtained for Siberian larch for the first time. The reference complete chloroplast genomes, such as one described here, would greatly help in the chloroplast resequencing and search for additional genetic markers using population samples. The results of this research will be useful for further phylogenetic and gene flow studies in conifers.

**Electronic supplementary material:**

The online version of this article (10.1186/s12859-018-2571-x) contains supplementary material, which is available to authorized users.

## Background

The chloroplast genome in conifers, including larch species [1], has a unique, strictly paternal inheritance via pollen, unlike angiosperms, where it has a maternal inheritance via seeds [2]. It allows tracing paternal gene flow and lineages separately from maternal (mitochondrial genes) and bi-parental (nuclear genes) ones. Therefore, chloroplast DNA sequences are the most important source of genetic markers to study distribution of paternal genes and paternally based molecular phylogenetic relationships in conifers.

Larch species, as well as many other conifer species are the main boreal forest tree species, which comprise ~ 30% of the world’s forested lands [3]. Boreal forests play a very important ecological role, but are also affected by the global climate change. On one hand, they suffer now from more frequent and drastic droughts, but on the other hand, their area is expanding in the northern regions, and their tree line is moving towards the north creating an ecotone, a highly dynamic transition area [4]. It is important to know how much of paternal associated gene flow by pollen contributes into establishing this zone, compared to the maternal and bi-parental contributions by seeds. Such studies require chloroplast markers. The next generation sequencing (NGS) technique allows whole chloroplast genome sequencing in multiple individuals and makes a search for molecular genetic markers more efficient. For instance, Parks et al. [5] sequenced chloroplast genomes in 37 pine species using NGS nearly completely. They found a significant amount of variation (especially in two loci *ycf1* and *ycf2*) that provided them with additional data for inferring intrageneric phylogeny of genus *Pinus*.

Whole chloroplast genome comparison across different species and genera allows also studying organelle evolution and how it is associated with speciation and dispersal. Complete chloroplast genome sequences are available in NCBI Genbank for multiple plant species, including conifers. However, most of them represent the *Pinus* genus, and only three chloroplast genomes are available for the *Larix* genus: complete - for European (*Larix decidua* Mill.; AB501189.1) and Chinese (*L. potaninii var. chinensis* Beissn.; KX808508) larch and partial - for Western larch (*L. occidentalis* Nutt.; FJ899578.1).

Variation in the chloroplast genome is effectively used in phylogenetics at different levels. It allowed discriminating different subgenera and genera. For instance, Cronn et al. [6] compared chloroplast genome sequences of seven pine and one spruce species and found three regions that have deletions corresponding to the subgenera specific deletions in three genes: *ycf12* (78 bp at the nucleotide starting position 51,051), *psaM* (93 bp at position 51,442), and *ndh*I (371 bp at position 101,988), respectively. These are common deletions in the chloroplast genome in pine species of the subgenus *Strobus* (i.e., *P. gerardiana, P. krempfii, P. lambertiana, P. longaeva, P. monophylla, P. nelsonii, P. koraiensis*); the corresponding genes were present in the subgenus *Pinus* (*P. contorta, P. ponderosa, P. thunbergii*) and in spruce *Picea sitchensis* [6].

Variation in the chloroplast genome can be also effectively used in discriminating different populations of the same species. For instance, Whittall et al. [7] demonstrated a strong differentiation between the mainland and island populations of Torrey pine (*Pinus torreyana*) based on 5 SNPs found in the entire chloroplast genome of 120 Kbp.

## Methods

We used the data of the whole genome sequencing of three Siberian larch trees generated by Illumina HiSeq2000 [8]. DNA samples were isolated from needles and haploid callus of three Siberian larch trees, representing different regions in Russia – the Ural Mountains, Krasnoyarsk Region and Khakassia Republic, respectively. *The Larix decidua* Mill. [9] and *L. occidentalis* Nutt. [5] chloroplast genomes were used as a reference (NCBI Genbank accession numbers AB501189.1 and FJ899578.1, respectively). We did not use the chloroplast genome of *L. potaninii* [10] as a reference, because it was assembled by using the chloroplast genome of *L. decidua* (NC_016058; [9]) as a reference, but we used it in the comparative analysis. The paired-end (PE) and mate-pair (MP) libraries with fragment sizes of 400–500 bp (Ural and Krasnoyarsk trees) and 300–400 bp (Khakassia tree), respectively, were used for sequencing via 2 × 100 cycles by Illumina HiSeq2000.

The sequence reads were mapped to the reference chloroplast genomes using the Bowtie2 software [11], which is good for mapping short sequence reads to medium-sized and large genomes. This software implements an algorithm to derive the FM-index based on the Burrows-Wheeler Transform. The SPAdes genome assembler has been used to assemble the larch genome, which implements the De Bruijn graph approach [12]. The Rapid Annotation service with Subsystem Technology (RAST) has been used for annotation [13].

The first step in our assembly procedure consisted of mapping short reads to the available chloroplast genome references of *L. decidua* and *L. occidentalis* using the Bowtie2 software. Then, the aligned reads were assembled by SPAdes. The obtained contigs were aligned again on the reference of *L. decidua* using BLAST. In the third step, the selected contigs were verified to get the “trusted” status. Then, the assembly was carried out using SPAdes. The final step of the assembly was scaffolding, which was done using the generated contigs and MP reads using the SSPACE program [14].

Considering the well-known fact that chloroplast organelle originated from cyanobacteria, and that, therefore, chloroplast genes are still very similar to the bacterial ones, the RAST service, which was designed for annotation of bacterial and archaeal genomes, was used for the larch genome annotation. The annotation obtained by the RAST contained both the confirmed known genes and the predicted genes, potentially coding hypothetical proteins. In order to clarify the roles of these hypothetical coding regions, our annotation was compared with the annotations of two closely related species, *L. decidua* and *L. occidentalis*, respectively. In addition, some fragments of the genome have been also selectively aligned with BLAST. The sites of hypothetical proteins confirmed by BLAST were identified and recorded.

The assembled chloroplast genome of *L. sibirica* has been deposited in the NCBI GenBank with the accession number NC_036811.1 and used as a reference to search for polymorphisms (SNPs and SSRs) among the three above mentioned trees. SNPs were searched using the Bowtie2 and UGENE [15] software (option *Call Variants with SAMtools*). First, the reads of the Urals and Khakassian trees were mapped to the finally assembled genome of the Krasnoyarsk tree. Then, the resulting *sam*-file together with the assembled genome was used by the UGENE program to search for SNPs. The SSR loci were searched using GMATo [16] with a threshold of minimum 6 repeats for di-, tri-, tetra-, penta-, and hexanucleotide motifs, and 10 minimum repeats for mononucleotide motifs.

## Results

The total length of the final Siberian larch chloroplast genome assembly was 122,560 bp, which is very close to 122,474 bp in the closely related European larch (*Larix decidua*). The annotation through comparison with the available data for *L. decidua* and *L. occidentalis* identified 110 genes, from which 34 represented tRNA genes, 4 rRNA, and 72 protein-coding genes. In three trees 13 SNPs were detected. Two of them were found in the coding regions of the *tRNA-Arg* and *Cell division protein ycf2* genes*.*

**Fig. 1:**
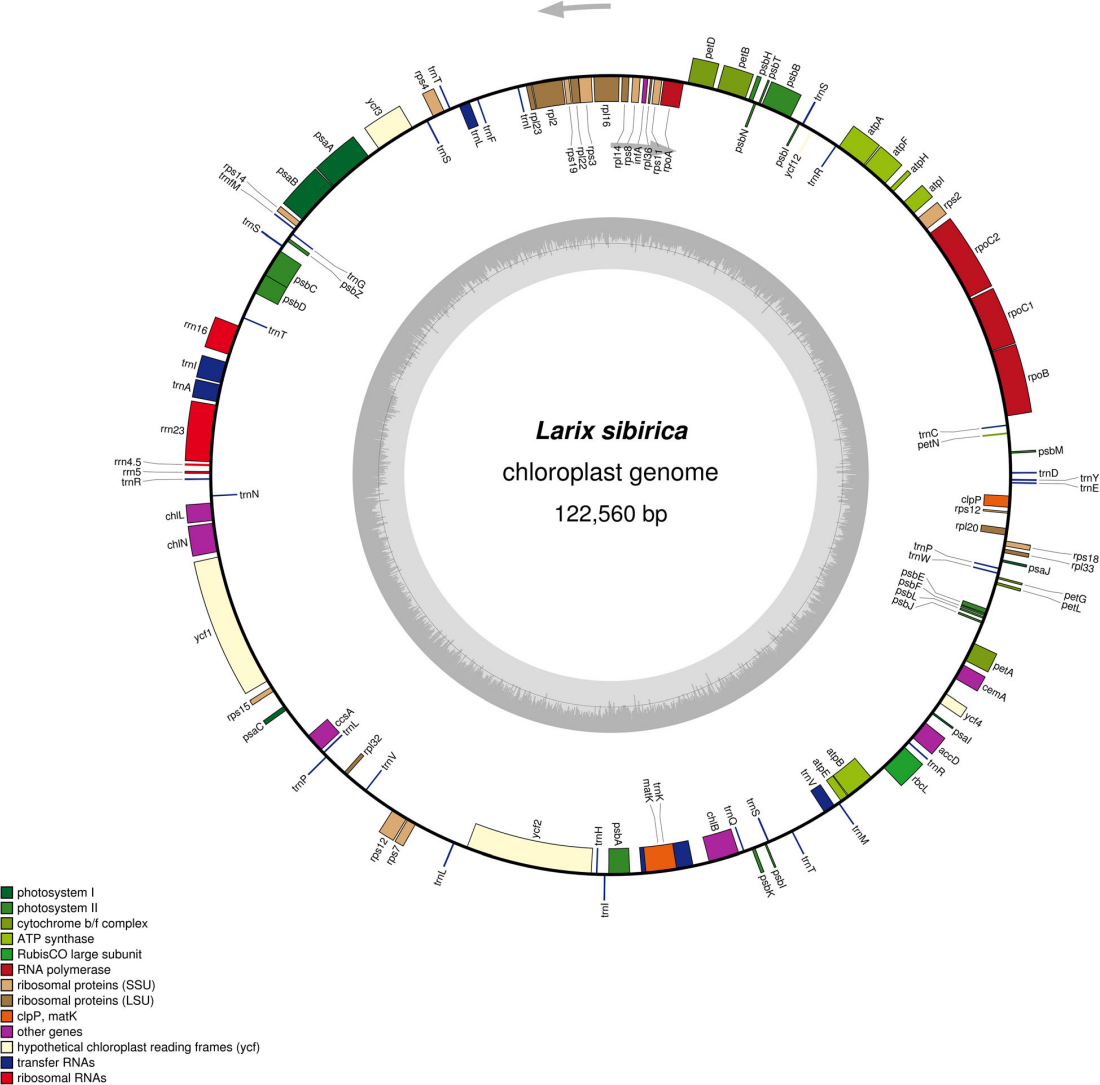
Gene map of the *Larix sibirica* chloroplast genome. Genes belonging to different functional groups are color-coded. The dark and light grey in the inner circle represents the GC and AT content, respectively

**Fig. 2:**
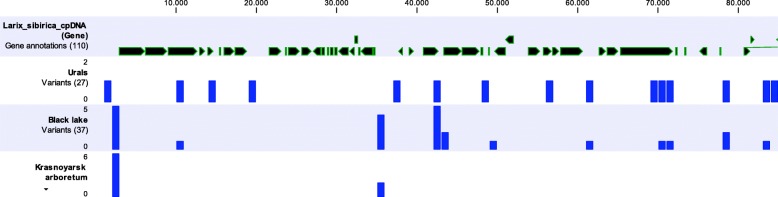
Variation detected in the *Larix sibirica* chloroplast genome (see also Additional file 1)

We used the available software, such as Bowtie2, BLAST, and SPAdes to assemble the chloroplast genome using the reads generated in the whole genome sequencing of the Siberian larch project. We used SSPACE for scaffolding and the RAST service for annotation of the obtained chloroplast genome. We developed a procedure that allowed us to successfully extract chloroplast genome specific reads and then assemble and annotate the resulting sequences. We identified and verified 110 coding regions representing 38 RNA and 72 protein genes, which is equal to the number of genes in chloroplast sequences of *L. decidua* and *L. potaninii* and close to 105 genes in the partial chloroplast genome sequence of *L. occidentalis*. A gene map of the genome was generated using OGDRAW [17] and is presented in Fig. 1. The search for SNPs using UGENE revealed a relatively small number of SNPs (Fig. 2; Additional file 1), but it is only preliminary data based on a limited sample size. In addition, 23 SSR loci (16 with mono- and 7 with dinucleotide repeat motifs) were identified in the chloroplast genome (Additional file 2). No SSR loci with tri-, tetra-, penta-, and hexanucleotide repeats were found with the search parameters used.

## Discussion

The chloroplast genome variation in most plants is often limited due to a relatively low frequency of mutations in this organelle. For example, the mutation rate of the chloroplast genome in pines is approximately 0.2–0.4 × 10^− 9^ synonymous substitutions per nucleotide per year [18, 19]. However, with an average length of 120–160 Kbp and 130 genes, the chloroplast genomes are sufficiently large and complex, and include structural and point mutations that reflect population differentiation and evolutionary divergence [6].

Unlike angiosperms, the conifer chloroplast DNA (cpDNA) lacks large inverted repeats (IR), but contains dispersed repetitive DNA that is associated with structural rearrangements. In addition to large dispersed repeated sequences, the conifer cpDNA also possesses a number of small repeats. It contains variable numbers of tandem repeats of 124 to 150 bp in size, which are associated with the polymorphic rearranged region near *trnK-psbA*, where the *psbA* gene has been duplicated [20].

Most variation in the chloroplast genome is associated with microsatellite loci [21, 22]. However, these markers have a too high mutation rate that can lead to incorrect phylogenetic inferences [23–25]. SNPs could be better markers for phylogenetic inferences, and comparative complete chloroplast genome studies are needed to discover these markers. The reference complete chloroplast genomes, such as the one described here, would greatly help in chloroplast resequencing and search for SNPs using population samples.

## Conclusions

The complete chloroplast genome sequence was obtained for Siberian larch for the first time. Annotation and comparison of the obtained data with data available only for two other larch species helped us identify and verify 110 coding regions representing 38 RNA and 72 protein genes. The total of 13 SNPs were detected; two of them were in the coding regions of the genome*.* The results of this research will be useful for further phylogenetic and gene flow studies in conifers.

## Additional files

Additional file 1: **Table S1.** Genetic variants identified in the Siberian larch chloroplast genome. (XLSX 24 kb)

Additional file 2: **Table S2.** Chloroplast microsatellite (SSR) identified in the Siberian larch chloroplast genome. (DOCX 14 kb)

## References

[1] Szmidt AE, Aldén T, Hällgren J-E (1987). Paternal inheritance of chloroplast DNA in *Larix*. Plant Mol Biol.

[2] Hipkins VD, Krutovskii KV, Strauss SH (1995). Organelle genomes in conifers: structure, evolution, and diversity. Forest Genet.

[3] Brandt JP, Flannigan MD, Maynard DG, Thompson ID, Volney WJA (2013). An introduction to Canada’s boreal zone: ecosystem processes, health, sustainability, and environmental issues. Environm Rev.

[4] Johnson JS, Gaddis KD, Cairns DM, Konganti K, Krutovsky KV (2017). Landscape genomic insights into the historic migration of mountain hemlock in response to Holocene climate change. Am J Bot.

[5] Parks M, Cronn R, Liston A (2009). Increasing phylogenetic resolution at low taxonomic levels using massively parallel sequencing of chloroplast genomes. BMC Biol.

[6] Cronn R, Liston A, Parks M, Gernandt DS, Shen R, Mockler T (2008). Multiplex sequencing of plant chloroplast genomes using Solexa sequencing-by-synthesis technology. Nucleic Acids Res.

[7] Whittall JB, Syring J, Parks M, Buenrostro J, Dick C, Liston A, Cronn R (2010). Finding a (pine) needle in a haystack: chloroplast genome sequence divergence in rare and widespread pines. Mol Ecol.

[8] Kuzmin DA, Feranchuk SI, Sharov VV, Cybin AN, Makolov SV, Putintseva YA, Oreshkova NV, Krutovsky KV. Stepwise large genome assembly approach: A case of Siberian larch (*Larix sibirica* Ledeb.). BMC Bioinformatics. 2019;20(Suppl. 1). 10.1186/s12859-018-2570-y. 10.1186/s12859-018-2570-y PMC6362582 30717661

[9] Wu CS, Lin CP, Hsu CY, Wang RJ, Chaw SM (2011). Comparative chloroplast genomes of Pinaceae: insights into the mechanism of diversified genomic organizations. Genome Biol Evol.

[10] Han K, Li J, Zeng S, Liu Z (2017). Complete chloroplast genome sequence of Chinese larch (*Larix potaninii* var. *chinensis*), an endangered conifer endemic to China. Conservation Genet Res.

[11] Langmead B, Salzberg S (2012). Fast gapped-read alignment with bowtie 2. Nat Methods.

[12] Bankevich A, Nurk S, Antipov D, Gurevich AA, Dvorkin M, Kulikov AS (2012). SPAdes: a new genome assembly algorithm and its applications to single-cell sequencing. J Comput Biol.

[13] Overbeek R, Olson R, Pusch GD, Olsen GJ, Davis JJ, Disz T (2014). The SEED and the rapid annotation of microbial genomes using subsystems technology (RAST). Nucleic Acids Res.

[14] Boetzer M, Henkel CV, Jansen HJ, Butler D, Pirovano W (2011). Scaffolding pre-assembled contigs using SSPACE. Bioinformatics.

[15] Okonechnikov K, Golosova O, Fursov M (2012). The UGENE team. Unipro UGENE: a unified bioinformatics toolkit. Bioinformatics.

[16] Wang X, Lu P, Luo Z (2013). GMATo: a novel tool for the identification and analysis of microsatellites in large genomes. Bioinformation.

[17] Lohse M, Drechsel O, Kahlau S, Bock R (2013). OrganellarGenomeDRAW – a suite of tools for generating physical maps of plastid and mitochondrial genomes and visualizing expression data sets. Nucleic Acids Res.

[18] Willyard A, Syring J, Gernandt DS, Liston A, Cronn R (2007). Fossil calibration of molecular divergence infers a moderate mutation rate and recent radiations for *Pinus*. Mol Biol Evol.

[19] Gernandt DS, Magallon S, Lopez GG, Flores OZ, Willyard A, Liston A (2008). Use of simultaneous analyses to guide fossil-based calibrations of Pinaceae phylogeny. Int J Plant Sci.

[20] Lidholm J (1991). Gustafsson P. a three-step model for the rearrangement of the chloroplast *trnK-psbA* region of the gymnosperm *Pinus contorta*. Nucleic Acids Res.

[21] Provan J, Soranzo N, Wilson NJ, Goldstein DB (1999). Powell W a. a low mutation rate for chloroplast microsatellites. Genetics.

[22] Ebert D, Peakall R (2009). Chloroplast simple sequence repeats (cpSSRs): technical resources and recommendations for expanding cpSSR discovery and applications to a wide array of plant species. Mol Ecol Res.

[23] Afzal-Rafii Z, Dodd A (2007). Chloroplast DNA supports a hypothesis of glacial refugia over postglacial recolonization in disjunct populations of black pine (*Pinus nigra*) in Western Europe. Mol Ecol.

[24] Höhn M, Gugerli F, Abran P, Bisztray G, Buonamici A, Cseke K (2009). Variation in the chloroplast DNA of Swiss stone pine (*Pinus cembra* L.) reflects contrasting post-glacial history of populations from the Carpathians and the Alps. J Biogeogr.

[25] Moreno-Letelier A, Piñero D (2009). Phylogeographic structure of *Pinus strobiformis* Engelm. Across the Chihuahuan Desert filter-barrier. J Biogeogr.

