# Supplementary material for: Siberian larch (Larix sibirica Ledeb.) chloroplast genome and development of polymorphic chloroplast markers
Source: BMC Bioinformatics. 2019 Feb 5;20(Suppl 1):38. doi: 10.1186/s12859-018-2571-x (PMC6362560; doi:10.1186/s12859-018-2571-x)
Supplement: Supplementary file 2 — Table S2. Chloroplast microsatellite (SSR) identified in the Siberian larch chloroplast genome. (DOCX 14 kb) [file 12859_2018_2571_MOESM2_ESM.docx]

**Table S2.** Chloroplast microsatellite (SSR) identified in the Siberian larch chloroplast genome.

| Start nucleotide position* | Motif and number of repeats* | Location |
| --- | --- | --- |
| 2727 | (T)_17_ | Non-coding region between *trnC* and *rpoB* genes |
| 3322 | (TA)_5_ | *rpoB* gene |
| 14848 | (A)_10_ | Non-coding region between *atpI* and *atpH* genes |
| 19001 | (T)_10_ | Non-coding region between *trnR* and *ycf12* genes |
| 19024 | (CT)_5_ | Non-coding region between *trnR* and *ycf12* genes |
| 19434 | (T)_11_ | Non-coding region between *trnR* and *ycf12* genes |
| 19523 | (T)_11_ | Non-coding region between *trnR* and *ycf12* genes |
| 31225 | (T)_10_ | *rpl16* gene |
| 32973 | (T)_13_ | Non-coding region between *rps19* and *rpl2* genes |
| 35993 | (G)_11_ | Non-coding region between *trnI* and *trnF* genes |
| 40518 | (T)_10_ | Non-coding region between *trnS* and *ycf3* genes |
| 42972 | (AT)_7_ | Non-coding region between *ycf3* and *psaA* genes |
| 48664 | (AT)_7_ | Non-coding region between *trnG* and *psbZ* genes |
| 65202 | (AT)_5_ | Non-coding region between *chlN* and *ycf1* genes |
| 71873 | (A)_13_ | Non-coding region between *ycf1* and *rps15* genes |
| 78982 | (A)_11_ | Non-coding region between *rpl32* and *trnV* genes |
| 80433 | (A)_11_ | Non-coding region between *trnV* and *rps12* genes |
| 83983 | (AT)_5_ | Non-coding region between *rps7* and *trnL* genes |
| 98910 | (C)_12_ | *psbK* intron region |
| 102867 | (AT_)8_ | Non-coding region between *trnT* and *trnV* genes |
| 118541 | (G)_11_ | *rps12* intron region |
| 120867 | (T)_15_ | Non-coding region between *rps12* and *clpP* genes |
| 121832 | (T)_10_ | Non-coding region between *clpP* and *trnE* genes |

* In the reference genome (NCBI GenBank accession number NC_036811.1)
